# Editorial: Training and Enhancing Executive Function

**DOI:** 10.3389/fpsyg.2020.02031

**Published:** 2020-08-28

**Authors:** Gian Marco Marzocchi, Maria Carmen Usai, Steven J. Howard

**Affiliations:** ^1^Department of Psychology, University of Milan-Bicocca, Milan, Italy; ^2^Department of Education, University of Genoa, Genoa, Italy; ^3^Early Start and School of Education, University of Wollongong, Wollongong, NSW, Australia

**Keywords:** executive functions, training, rehabilitation, cognitive control, technological interventions

Executive Function (EF) refers to a complex set of cognitive control processes necessary for adaptive functioning in daily life. EFs are predictive of intellectual achievement, health, wealth, and quality of life across the entire life span (Moffitt et al., 2011), often more so than IQ or socioeconomic status (Bertollo and Yerys, 2019). Evidence suggests that EFs can be distinguished into three core capacities (working memory, inhibition, and shifting), which combine to support the higher-order cognitive processing (e.g., planning, problem solving) required to remain goal-directed, resist contrary impulses and distraction, and pursue more-positive (rather than most-immediate) outcomes. Given this foundational importance, there has been increasing interest in improving EFs. The goals of this line of research have been 2-fold: to improve EFs; and, as a consequence, stimulate generalized improvements to other cognitive and life domains.

Yet approaches to stimulate EF change are highly discrepant, have yielded inconsistent effects and limited transfer to untrained abilities and outcomes, and there is little agreement about the underlying mechanisms of change that are needed to stimulate development in EFs (and EF-related trajectories and outcomes). This special issue thus aimed to provide a snapshot of current evidence, approaches and perspectives on EF intervention, to highlight emerging insights, to stimulate a reconciliation, and to identify a unified way forward. Typifying contemporary investigations in this field, we characterize submissions to this special issue as falling into one of five approaches for fostering EFs: play-based and curricular approaches; technology-leveraged approaches; physical approaches; strategy-based approaches; and investigations of plausible causal mechanisms. In our conclusion, we offer some of our take-aways and suggestions for a possible way forward in this area.

## Play-Based and Curricular Approaches

The largest category of submissions to this special issue pertained to play-based and/or curricular approaches to fostering EFs with pre-school and primary school children. In this approach, everyday playful situations and activities were used to inject EF challenge into real-world situations, with the aim of promoting EFs and related abilities (e.g., school readiness, learning). For instance, the PRSIST Program (Howard et al.), Red Light Purple Light Circle Time Games (McClelland et al.), and Chicco and Nana (in the Italian version; Traverso et al.) supported pre-school educators to engage young children in EF-injected activities and games. Each program generated small improvement in EFs, with the latter two also generating gains in academic learning when curricular content was added. Similar EF gains were found amongst primary school students 8 months after completion of a school-based program of EF games and activities (Rosas et al.).

Other studies within this category integrated EF challenge into school curricula and activities. For instance, the PENcE program (de Oliveira Cardoso et al.) yielded EF benefits after teacher-led instruction of EF strategies, which were practiced during in-school activities and then extended to real-world situations. Teacher-rated EFs also improved after an arts- and culture-focused program that injected EF challenge into music, drama, dance, literature, art, and/or photography lessons (Andersen et al.), and objectively measured EFs similarly improved after a pre-school music program (Shen et al.).

While we consider these as play-based and curricular approaches, of course alternative classifications are possible and there is substantial overlap between these and subsequent studies. In fact, many of these programs also included technology, motor activities, and/or strategy-based instruction that are central to approaches otherwise classified. This highlights the likelihood of a set of general principles of effective EF intervention, which may need to be adjusted to align with the “what, where, who, why, and how” of EF intervention.

## Technology-Leveraged Approaches

Several studies used a technology-leveraged approach to improve EFs across different populations, including typically developing, special needs, and preterm children from pre-school- to school-age. Although we characterized five studies as technology-leveraged intervention, we can distinguish two distinct approaches to technology use amongst them. In one case, the technological tools were used to deliver the intervention. This is the case of the intervention presented in van Houdt et al.'s study, where the BrainGame Brian Training sought to improve EF abilities in a randomized controlled trial involving school-aged children born very preterm. Another example is Rossignoli-Palomeque et al.'s study, in which the Nexxo-training app was supplemented by metacognitive strategies given by an instructor to improve inhibition and vigilance abilities. Technology was also employed as a vehicle for the delivery of Exergames, where body movements were necessary to play a computer game through connected devices (Mossmann et al.). Consistent amongst these approaches was their engagement and challenge of EFs through effortful tasks (Diamond and Ling, 2016), although EF improvements were inconsistent and there were limited generalized improvements in untrained and real-world outcomes.

The other approach, which in the current set of studies seemed to be more promising in terms of EF gains, was when a technology-based intervention exploited use of computational processes to perform activities in the real world, such as with the educational robotics approach (Di Lieto, Castro et al.; Di Lieto, Pecini et al.). That is, educational robotics uses programming principles that are used to manipulate concrete objects (i.e., robots) to speculate and test effects of this manipulation. In doing so, these approaches seek to promote not only academic skills, but also domain-general skills such as planning, problem-solving and metacognitive abilities. In our compilation, this approach yielded effects in both school- and pre-school-aged children, and with special needs groups. A similar approach, based on computational thinking and code processing activities (Arfé et al.), yielded significantly improved planning and inhibition skills.

Taken together, these results suggest that technology can provide a virtual environment to stimulate EF challenge, but that this can be more or less effective in promoting EF and in generalizing benefit to real-world behavior. More positive results seemed to drive from interventions in which technology compelled children to engage EFs in activities played outside of a virtual environment.

## Physical Approaches

Another approach sought to leverage the physical body to promote EF. Several possible mechanisms for such an effect have been suggested. Singh and Mutreja's study explored the mechanisms through which specific body activities might influence EF, specifically investigating the association of postural and breath control with EF, through Yoga training. The authors suggest that attention to postural and breath control during yoga asanas and pranayama, respectively, may play a key role in enhancing EF. Inhibition was shown to benefit from yoga training, in particular, compared to working memory or shifting.

## Strategy-Based Approaches

In contrast to previous studies that target expansion of EF capacities, Chan et al. adopted a strategy-promotion approach to improve effective and efficient use of EFs. This approach provides participants with explicit instruction and practice to improve strategies useful in situations where it is necessary to exercise EF control. In this study, children became able to generalize and apply different strategies to novel problem-solving tasks, on which they did not receive any explicit instruction.

## Causal Mechanisms Investigation

Another promising approach to investigate the causal mechanisms for promoting EFs is represented by Perry et al.'s contribution, in which a rat model was employed to investigate the extent to which social experiences and the acquisition of social skills contribute to the development of EFs. Although it is unclear to what extent these findings can be extended to human situations, it represents an experimental confirmation with rats of negative impacts of early adverse conditions and the role of social relations as a moderator of EF development.

## Conclusions

An average effect of EF training with preschoolers was provided by Scionti et al.; they found small EF improvement, probably due to young age of the participants and weak specialization of their EFs. Moreover, they found that these programs were significantly more effective for developmentally at-risk children (e.g., ADHD, low SES) than for typically developing children.

In conclusion, we propose that future studies on EF training should integrate, comparatively evaluate, and reconcile across different approaches (play and curriculum based, leveraging new technologies) to discern principles for effective EF intervention. These approaches should stimulate both cognitive and emotional-motivational aspects of EF to increase likelihood of far transfer to real-world outcomes of interest (e.g., academic and life success). Finally, EF studies should pay particular attention to what works for whom, and under what conditions.

## Author Contributions

All authors listed have made a substantial, direct and intellectual contribution to the work, and approved it for publication.

## Conflict of Interest

The authors declare that the research was conducted in the absence of any commercial or financial relationships that could be construed as a potential conflict of interest.
